# Bi-Allelic Pathogenic Variations in *MERTK* Including Deletions Are Associated with an Early Onset Progressive Form of Retinitis Pigmentosa

**DOI:** 10.3390/genes11121517

**Published:** 2020-12-18

**Authors:** Cathrine Jespersgaard, Mette Bertelsen, Farah Arif, Helene Gry Gellert-Kristensen, Mingyan Fang, Hanne Jensen, Thomas Rosenberg, Zeynep Tümer, Lisbeth Birk Møller, Karen Brøndum-Nielsen, Karen Grønskov

**Affiliations:** 1 Kennedy Center, Department of Clinical Genetics, Rigshospitalet, University of Copenhagen, 2600 Glostrup, Denmark; CJE@ngc.dk (C.J.); Mette.bertelsen@regionh.dk (M.B.); Helene.gry.gellert-kristensen.01@regionh.dk (H.G.G.-K.); Asuman.zeynep.tuemer@regionh.dk (Z.T.); Lisbeth.birk.moeller@regionh.dk (L.B.M.); karen.broendum-nielsen@regionh.dk (K.B.-N.); 2 Danish National Genome Center, 2300 Copenhagen, Denmark; 3 Department of Ophthalmology, Rigshospitalet-Glostrup, University of Copenhagen, 2600 Glostrup, Denmark; Farah.arif@regionh.dk (F.A.); Hanne.Jensen.02@regionh.dk (H.J.); tro@eyenet.dk (T.R.); 4 BGI-Shenzhen, Shenzhen 518083, China; Fangmingyan@genomics.cn

**Keywords:** retinitis pigmentosa, *MERTK*, CNV

## Abstract

Bi-allelic pathogenic variants in *MERTK* cause retinitis pigmentosa (RP). Since deletions of more than one exon have been reported repeatedly for *MERTK*, CNV (copy number variation) analysis of next-generation sequencing (NGS) data has proven important in molecular genetic diagnostics of *MERTK*. CNV analysis was performed on NGS data of 677 individuals with inherited retinal diseases (IRD) and confirmed by quantitative RT-PCR analysis. Clinical evaluation was based on retrospective records. Clinical re-examination included visual field examination, dark adaption, scotopic and photopic full-field electroretinograms (ffERG), multifocal ERG (mfERG) and optic coherence tomography (OCT). Fourteen variants were detected in *MERTK* in six individuals, three of which were deletions of more than one exon. Clinical examinations of five out of six individuals revealed a severe phenotype with early-onset generalized retinal dystrophy with night blindness and progressive visual field loss; however, one individual had a milder phenotype. Three individuals had hearing impairments. We show that deletions represent a substantial part of the causative variants in *MERTK* and emphasize that CNV analysis should be included in the molecular genetic diagnostics of IRDs.

## 1. Introduction

Inherited retinal diseases (IRD) encompass a broad range of clinical diagnoses characterized by the progressive loss of photoreceptors, with variable ages of onset and variable clinical representation. IRD is highly genetically heterogeneous with more than 250 associated genes (https://sph.uth.edu/retnet/), which show all modes of Mendelian inheritance (autosomal dominant, autosomal recessive, X-linked), mitochondrial inheritance, and even digenic inheritance [1]. The majority of the genes encode proteins exerting their function in either the photoreceptor or in the retinal pigment epithelium (RPE) cells. They are involved in various cellular processes such as RNA splicing, visual cycle, primary cilia function and phototransduction [2]. Retinitis pigmentosa (RP) is the most frequent of the IRD specific diagnostic subgroups with a worldwide prevalence of 1:3500 [3]. RP is characterized by dysfunctional rod photoreceptors in the early stages of disease, causing night blindness; but eventually, the cone photoreceptors may also be affected, leading to visual field constriction and central vision loss [2].

More than 120 genes are known to be associated with RP [2] and *MERTK* (MER tyrosinase kinase protooncogene) is associated with autosomal recessive RP type 38 (OMIM 613862) [4]. A recent review shows that the *MERTK* associated RP comprises around 2% of RP [5]. *MERTK* is located at 2q14.1 and consists of 19 exons encoding a receptor tyrosine kinase of 999 amino acids belonging to the TAM (TYRO3/AXL/MER) receptor family [6]. It is highly expressed on the apical membrane of the RPE cells as well as in the monocytes/macrophages, ovary, prostate, testis, lung and kidney [7]. *MERTK* is involved in the internalization of the photoreceptor outer segment (POS) prior to phagocytosis in RPE [8]. *MERTK* ligands GAS6 (growth arrest-specific protein 6) and PROS1 (protein S) is secreted by the RPE cells and binds to phosphatidylserines (PS) on the surface of POS and to *MERTK* on the surface of RPE cells; this leads to *MERTK* phosphorylation and activation of phagocytosis of POS of RPE cells [9,10]. Pathogenic variants in *MERTK* cause a severe RP phenotype with early age of onset and early involvement of the macular region, very often leading to blindness. The disease is characterized by night blindness in the childhood or teens, abnormal color vision, reduced visual acuity and visual field constriction [4].

More than 90 disease-causing or probably disease-causing variants are reported in HGMD^®^ Professional (2020.3), including nine gross deletions, 29 missense variants, 15 nonsense variants, 16 splicing variants, 16 small deletions, three small insertions and three small indels [11]. A founder variant of *MERTK* (deletion of exons 1 to 7) was found in the Faroe Islands and is responsible for approximately 30% of all RP cases within the Faroese population [12].

Copy number variations (CNV) has become an important contributor to the cause of retinal dystrophies [13,14,15].

We report the molecular genetic findings in *MERTK* and the clinical representation in six individuals as part of a large screening study of 677 individuals with a clinical diagnosis of IRD [16].

## 2. Materials and Methods

### 2.1. Editorial Policies and Ethical Considerations

The project was approved by The National Committee on Health Research Ethics, Denmark (project ID 1301394, 1418960 and 1809595). The project was performed according to the Declaration of Helsinki and approved by the Regional Ethics Committee. Written informed consent was obtained before the molecular genetic testing.

### 2.2. Clinical Evaluation

Clinical examinations were performed by the Eye clinic at the Kennedy Center, Rigshospitalet (former National Eye Clinic for the Visually Impaired). The clinical evaluation was based on available retrospective clinical records with information on clinical diagnosis, clinical history, fundus changes, OCT imaging, slit lamp examination, ERG, visual acuity and Goldmann visual fields. Furthermore, P152, P155, P156 and affected brother of individual P152 were re-examined after the genetic diagnosis was made.

### 2.3. Molecular Genetic Analysis

Genomic DNA of 677 individuals with IRD who was sequenced with targeted NGS of 125 genes as described previously [16] (Table S1). In brief, the enriched DNA libraries were sequenced using the Illumina HiSeq 2000 (San Diego, CA, USA). Raw sequencing image files and base-calling were processed with the Illumina Pipeline and raw paired-end low quality reads and adapter sequences were removed using the SOAPnuke software (https://bio.tools/soapdenovo). The remaining high-quality reads were aligned to the human reference genome (GRCh37/hg19) using Burrows–Wheeler Algorithm (BWA) version 0.7.1023, with the MEM algorithm. The SAMtools (version 0.1.19) [17] was used to sort and index SAM/BAM files and the Picard (version 1.117, http://broadinstitute.github.io/picard/) was used to mark PCR-duplicates. Local realignment and base recalibration were performed using GATK (version 3.3-0) [18] and single nucleotide variants (SNVs) and insertions/deletions (InDels) were called with GATK HaplotypeCaller.

CNV analysis for the data was carried out by using the ExomeDepth algorithm based on the coverage data [19]. For each tested individual, the ExomeDepth algorithm builds the most appropriate reference set from the BAM files of a group of samples and ranks the CNV calls by their confidence level. A subset of samples was analyzed using the CNV algorithm in Varseq (Golden Helix, Bozeman, MT, USA). The Varseq software generates a set of matched reference controls and the sample is compared to this set. A ratio and z-score are computed for each region defined in the BED file defining the targeted region. The z-score indicate the number of standard deviations that a sample’s coverage is from the mean coverage of the reference set. CNVs were verified with quantitative RT-PCR (qPCR) using SYBR green and analysis on a 7500 ABI SDS system (Applied Biosystems, Foster City, CA, USA). Breakpoint mapping of the deletion including exon 1 to exon 7 was performed using a standard PCR method for amplification of fragment spanning the breakpoint followed by Sanger sequencing. Primers were designed assuming the breakpoint was in the same region as the deletion found by Ostergaard et al. [12]. Primer sequences for qPCR and breakpoint mapping are listed in Table S2.

Variants were classified according to ACMG guidelines [20] and ACGS guidelines [21]. The CADD score was calculated for missense variants [22]. Variants in splice sites were evaluated using SpliceSiteFinder-like [23,24], MaxEntScan [25], NNSPLICE [26], GeneSplicer [27].

## 3. Results

### 3.1. Clinical Characteristics

**Figure 1:**
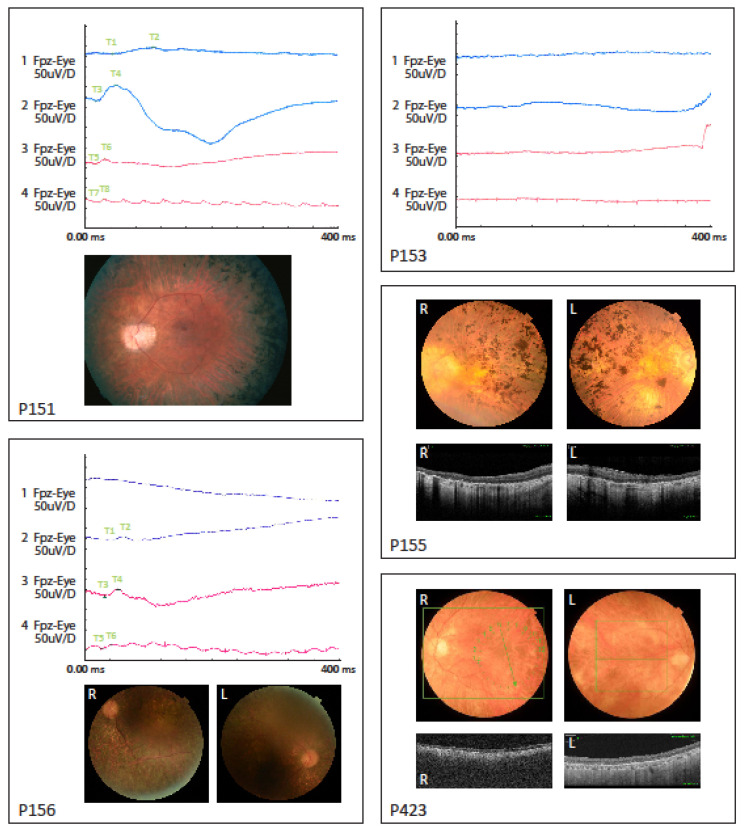
Electroretinograms (ERG) data shown for P151, P153 and P156. Fundus pictures shown for P151, P155, P156 and P423. OCT pictures for P155 and P423. Descriptions of data are listed in Table 1.

**Figure 2:**
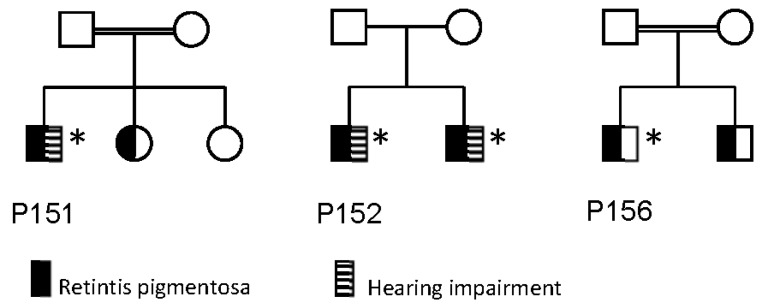
Pedigrees of families of P151, P152 and P156. Individuals marked with an asterix (*) has been genotyped, and has the genotype listed in Table 2.

Clinical data are presented in Table 1 and Figure 1. Pedigrees for three families are shown in Figure 2.

In general, the fundoscopic changes were characterized by a pale optic nerve, attenuated retinal vessels, peripheral retinal degeneration and bone spicule hyperpigmentation. Five out of the six individuals showed fundus and/or OCT signs of macular atrophy. The OCT analysis showed severe retinal degeneration and abnormal structure. In two individuals (P152 and P153) the retinal dystrophy was already generalized and severe when full-field ERG was performed, resulting in undetectable responses. This was also seen in the brother of P152. In P151, P156 and P423, earlier full-field ERG measured at disease onset were available and showed reduced rod and cone responses with primary loss of rod function. Additionally, all individuals had night blindness as the initial symptom consistent with the clinical classification of rod-cone dystrophy or RP.

Three individuals (P151, P152 and P155) were diagnosed with hearing impairment. P152 and his brother were diagnosed with a progressive bilateral hearing loss in the higher frequencies (2–4 KHz) diagnosed in their mid-thirties. P151 and P155 had anamnestically unspecific hearing loss in older age, with no hearing curves available.

### 3.2. Genetic Analysis

**Figure 3:**
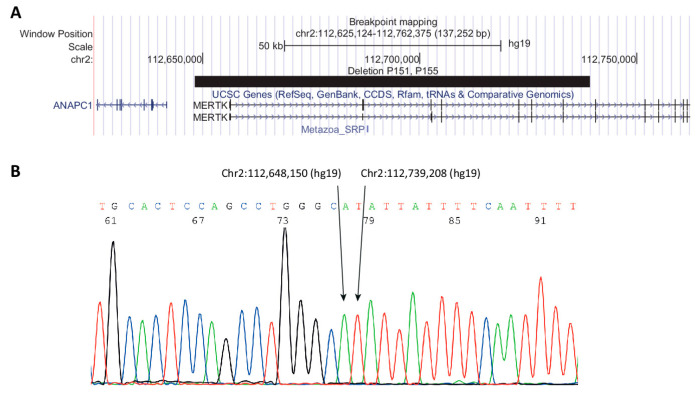
Breakpoint mapping of deletion in P151 and P155. (**A**) UCSC (University of California Santa Cruz) browser track showing deletion as a black bar. (**B**) Sanger sequencing showing the sequence surrounding the breakpoint.

Fourteen variants (seven unique variants) were found in six individuals (Table 2). Three variants were the deletion of exons 1 to 7 (P151 and P155). Breakpoint mapping showed that the breakpoints were identical in P151 and P155 and to the breakpoint mapped by Ostergaard et al. [12] (Figure 3). The deletion includes chr2:112,648,150-112,739,208 (GRCh37/hg19). Results from qPCR analysis are shown in Figure S1.

Of the seven unique variants, five had not previously been reported in individuals with retinal dystrophy (c.960+1G>A, c.757+1G>A, c.2060G>T and c.2305A>G). Two of the variants, c.2060G>T; p.(Arg687Leu) and c.2305A>G; p.(Ile769Val)), were found in the same individual (P423). Both variants were apparently homozygous and both were classified as variants of unknown significance (VUS). The variants c.960+1G>A and c.757+1G>A are both splice variants predicted to alter RNA splicing.

## 4. Discussion

In this study, we analyzed *MERTK* as a part of a targeted sequencing panel in individuals with IRD. Variants in *MERTK* were found in six individuals of which six had a potential molecular genetic diagnosis caused by variants in *MERTK*. These individuals were part of a cohort of 677, of which 421 had a clinical diagnosis of RP, corresponding to 1.7% of individuals with RP being caused by variants in *MERTK*, which is comparable to the findings of Audo et al. [5]. A total of 24 CNVs were found in the cohort of 677 individuals (3.5%) and of these four were in *MERTK*; only EYS with seven CNVs had more deletions/duplications than *MERTK* [16]. Others have performed CNV analysis using NGS data and found substantially higher yields of causal CNVs [13,15]. However, their numbers are based on unsolved cases. This underscores the importance of CNV analysis in IRDs and reflects that missing genetic explanations of IRDs can be found in already known IRD genes, as structural variants and deep intron variants affecting splicing. Thus, robust bioinformatic pipelines analyzing data from NGS targeted panels (and soon also data from whole-genome sequencing) are necessary, as are methods to detect deep intronic variants (for example RNA seq).

Most individuals had a severe phenotype with an onset of night blindness in the first decade of life and progressive visual field loss during childhood. Individual P423 however, presented with a milder phenotype. P423 had a late-onset (age 53 years) of night blindness and a slow progression of visual acuity and subsequent visual field loss. None of the individuals in our cohort had nystagmus. P423 belongs to a large family with several members affected with RP, however, the closest affected relative was a sister of her paternal grandmother and the affected descendants span three generations resembling autosomal dominant inheritance. Testing of two affected family members showed that these did not have the missense variants in *MERTK* found in P423. Either the missense variants (both classified as VUS) are not the genetic cause of IRD in P423, or there are two different IRDs in the family, one type explained by *MERTK* variants and another with a yet unknown genetic cause.

The deletion of exon 1–7 (Chr2:112,648,150-112,739,208 (hg19)) of *MERTK* was first reported by Ostergaard et al. [12] as a common founder variant in The Faroe Islands. The deletion has since been found in individuals outside The Faroe Islands by Ellingford et al. [32] in a study from Manchester Royal Eye Hospital, and in two individuals in this study (P152 and P155); it is unclear whether the deletion is a hotspot or a founder variant.

The ophthalmological findings in the individuals in this study are consistent with previous descriptions of individuals with *MERTK*-related retinal dystrophy [5]. Individuals with *MERTK*-related retinal dystrophy have previously been described both with rod-cone dystrophy and cone-rod dystrophy [5]. In our cohort, the phenotype of all individuals was clinically classified as rod-cone dystrophy with night blindness as the first symptom and verified by full-field ERG with a primary rod dysfunction in four individuals. One individual in our cohort, however, presented with a milder phenotype with later onset and milder disease, but as discussed above the molecular genetic diagnosis in this individual is questionable, although a broadened clinical spectrum of *MERTK*-related retinal dystrophy cannot be excluded. Clinical variety in terms of disease severity is a known phenomenon in individuals with RP with many other genetic causes [33]. The clinical variety may in part be explained by the type of variant involved, but there may also be additional factors both genetic, epigenetic and environmental factors that influence the phenotype.

To our knowledge hearing impairment has not been reported as part of the *MERTK*-related phenotype. Individuals P151 and P155 had late-onset hearing loss while the hearing loss in P152 and his brother was of another type. This points to the hearing loss in these individuals might as well be coincidental, as a result of other genetic or non-genetic causes.

In conclusion, this study adds to the understanding of the genetic and clinical characteristics of individuals with *MERTK*-related retinal dystrophies and underlines that CNV analysis should be added to the genetic evaluation of individuals suspected of having retinal dystrophy. New treatments such as gene therapy for retinal dystrophies are evolving and the understanding of the clinical characteristics of genotype-specific disease entities is important.

## Figures and Tables

**Table 1:** Clinical symptoms.

ID	Sex (M/F)	Clinical Diagnosis	Decade First Symptoms	Clinical History	First OPH Exam, Year (Age)	Latest OPH Exam, Year (Age)	BCVA OD/OS First Visit (Age)	BCVA OD/OS Last Visit (Age)	Refractive Error OD/OS First Visit	Refractive Error OD/OS Last Visit	VF First Exam (Age)	VF Latest Exam (Age)	(Phph)	Colour Vision (Age)	Slit Lamp (Age)	Dark Adoptometry (Age)	ERG (Age)	Latest Fundoscopy (Age)	Latest OCT (Age)	Other Symptoms/Signs
151	M	RP	1st	At age 7 poor night vision and peripheral visual field loss. Slow progression	1964 (9)	1991 (36)	0.67/0.80 (9)	0.6/0.6 (36)	−4.5−1.00 × 0/−4.5−1.00 × 0	−8.5−2.00 × 170/−7.75−1.75 × 170	Normal (ad modum Donders) (11)	Both eyes constricted to 20° (36)	1	ND	Normal (36)	Normal (36)	Reduced responses, rod-cone dysfunction (36)	Normal optic nerve, peripapillar atrophy, normal macula, attenuated retinal vessels, bone spicule hyperpigmentation in the periphery (36)	N/A	Mild hearing impairment
152	M	RP	1st	First symptoms were night blindness and during early childhood progressive constrictive visual field and colour vision defects. Visual acuity 1/60 both eyes and a visual field of only a few degrees at the age of 24 years	1971 (17)	2016 (62)	0.40/0.40 (17)	no LP (62)	−1.50−1.50 × 80	−1.50−0.50 × 90	Both eyes contricted to 5° (17)	No visual field (62)	2	Ishihara only errors both eyes (17)	Right eye: aphacic (phaco, 2016). Left eye: moderate nuclear and subcapsular cataract (62)	N/A	Undetectable (30)	Pale atrophic optic disc, attenuated vessels, severe generalized retinal degeneration with numerous hyperpigmentation both central and in the periphery (62)	Severe retinal degeneration and abnormal structure (62)	Hearing impairment, chronic lymphatic leukemia and hypertension
153	M	RP	1st	First symptoms of night blindness and photophobia at age 7. Thereafter slow progression of visual acuity and visual field loss	1984 (21)	2003 (40)	0.33/0.33 (21)	0.10/0.10 (40)	+2.00−0.50 × 100	+1.00−0.50 × 80	Slightly constricted (ad modum Donders) (21)	Both eyes constricted to 5° (40)	1	N/A	Normal (40)	Delayed and final threshold elevated approximately 2 log units above normal level (30)	Undetectable (30)	Pale atrophic optic disc with peripapillary drusen. Attenuated vessels, severe generalized retinal degeneration with peripheral bone spicule hyperpigmentation (38)	N/A	No
155	M	RP	1st	Since childhood decreased central vision and night vision. Slow progression of visual field loss	1973 (17)	2018 (61)	0.10/0.10 (17)	LP/LP (61)	−5.00/−4.00	+0.25−1.50 × 170/−0.25-1.25 × 172	Both eyes: constricted to 1–2°, preserved temporal island (17)	Cannot cooperate to visual field test (61)	1	ND	Cataract operation in 2016, otherwise normal (61)	ND	ND	Waxy optic disc pallor, attenuated retinal vessels, pigment deposits in the macula, bone spicule hyperpigmentation in periphery (61)	Right eye: macular atrophy. Left eye: epiretinal membrane, macular atrophy and edema (61)	Hearing impairment since 2015
156	M	RP	2nd	Since early teens decreased central vision and poor night vision. Slow progression of visual field loss	1993 (22)	2017 (46)	0.50/0.25 (22)	0.04/0.03 (46)	−0.25−1.75 × 10/−0.25−2.00 × 10	+1.00−1.50 × 120/+0.50−0.75 × 175	Both eyes: constricted to 20° (22)	Both eyes constricted to 5° (46)	1	Affected (22)	Incipient cataract (46)	Monophasic curve with loss of rod mediated dark adaptation (22)	Reduced responses, rod-cone dysfunction (22)	Pale optic nerve, attenuated retinal vessels, macular atrophy, peripheral bone spicule hyperpigmentation (22)		No
423	F	RP	6th	Late onset with night blindness at the age of 53 years. Slow progression of visual field loss thereafter	2005 (53)	2015 (62)	0.6/0.8 (53)	0.30/0.50 (62)	−1.00−1.25 × 155/−0.5−0.5 × 0	−0.50−0.50 × 0 (before cataract operation)	Normal (53)	Both eyes constricted to 5° and preserved inferior islands of 10 × 30°(62)	1–2	Slight dyschromatopsia (53)	Normal (53)	Monophasic curve with loss of rod mediated dark adaptation (53)	Rod response undetectable and cone response reduced and with prolonged implicit time (53)	Optic disc pallor and slightly attenuated vessels. Generalized light retinal coloring. No hyperpigmentation (53)	N/A	Thrombocytopenic purpura, rheumatoid arthritis and mixed connected tissue disease

M: Male; F: Female; BCVA: Best-corrected visual acuity; OD: Oculus dexter; OS: Oculus sinister; Phph: Photophobia; VF: Visual field; LP: Light perception; ND: not determined; ERG: Electroretinography; OCT: Optic coherence tomography; OPH: Ophthalmological.

**Table 2:** Molecular genetic findings in *MERTK.*

ID	cDNA^1^	Predicted Protein Change	AF in gnomAD	CADD Score	SSF/MES/NNS/GS	Class	ClinVar ID	Previously Reported
P151	c.345C>G	p.(Cys115Trp)	12/281826/0	22.9	NA	VUS (PM2, PS4(mod), PP3)	RCV000787624.1	[28,29]
	c.345C>G	p.(Cys115Trp)	12/281826/0	22.9	NA	VUS (PM2, PS4(mod), PP3)	RCV000787624.1	[28,29]
P152 (+brother)	chr2:112,648,150-112,739,208del (hg19)	NA	1/21694/0	NA	NA	Pathogenic (PVS1, PM2, PP1)	VCV000636043.1	[12,30]
	chr2:112,648,150-112,739,208del (hg19)	NA	1/21694/0	NA	NA	Pathogenic (PVS1, PM2, PP1)	VCV000636043.1	[12,30]
P153	c.960+1G>A	p.?	NP	NA	-/-/-/-	Likely pathogenic (PVS1, PM2)	RCV000787626.1	[16]
	c.960+1G>A	p.?	NP	NA	-/-/-/-	Likely pathogenic (PVS1, PM2)	RCV000787626.1	[16]
P155	c.757+1G>A	p.?	NP	NA	-/-/-/-	Likely pathogenic (PVS1, PM2)	RCV000787625.1	[16]
	chr2:112,648,150-112,739,208del (hg19)	NA	1/21694/0	NA	-/-/-/-	Pathogenic (PVS1, PM2, PP1)	VCV000636043.1	[12,30]
P156	c.1450G>A	p.(Gly484Ser), splice variant?	3/251408/0	25.3	NA	VUS (PM2, PS4(sup))	RCV000132663.2	[28,31]
	c.1450G>A	p.(Gly484Ser), splice variant?	3/251408/0	25.3	NA	VUS (PM2, PS4(sup))	RCV000132663.2	[28,31]
P423	c.2060G>T;2305A>G	p.(Arg687Leu;Ile769Val)	NP; 92/282478/0	32/19.01	NA	VUS (PM2, PP3)	RCV000787849.1/RCV787914.1	Novel
	c.2060G>T;2305A>G	p.(Arg687Leu;Ile769Val)	NP; 87/276840/0	32/19.01	NA	VUS (PM2, PP3)	RCV000787849.1/RCV787914.1	Novel

NA: not applicable; NP: not present; AF: allele frequency. MAF are presented as total number of alternate allele/total number of alleles/homozygous individuals. VUS: variant with unknown clinical significance; SSF: SpliceSiteFinder-like; MES: MaxEntScan; NNS: NNSPLICE; GS: GeneSplicer. *MERTK* transcript: NM_006343.2. ^1^ It was not possible to investigate the phase of the variants.

## Supplementary Materials

The following are available online at https://www.mdpi.com/2073-4425/11/12/1517/s1, Figure S1: qPCR analysis and BMA alignment. Table S1: Genes in gene panel. Table S2: Primers used for qPCR.
