# Supplementary material for: Bi-Allelic Pathogenic Variations in MERTK Including Deletions Are Associated with an Early Onset Progressive Form of Retinitis Pigmentosa
Source: Genes (Basel). 2020 Dec 18;11(12):1517. doi: 10.3390/genes11121517 (PMC7766129; doi:10.3390/genes11121517)
Supplement: Supplementary file 1 [file genes-11-01517-s001.pdf]

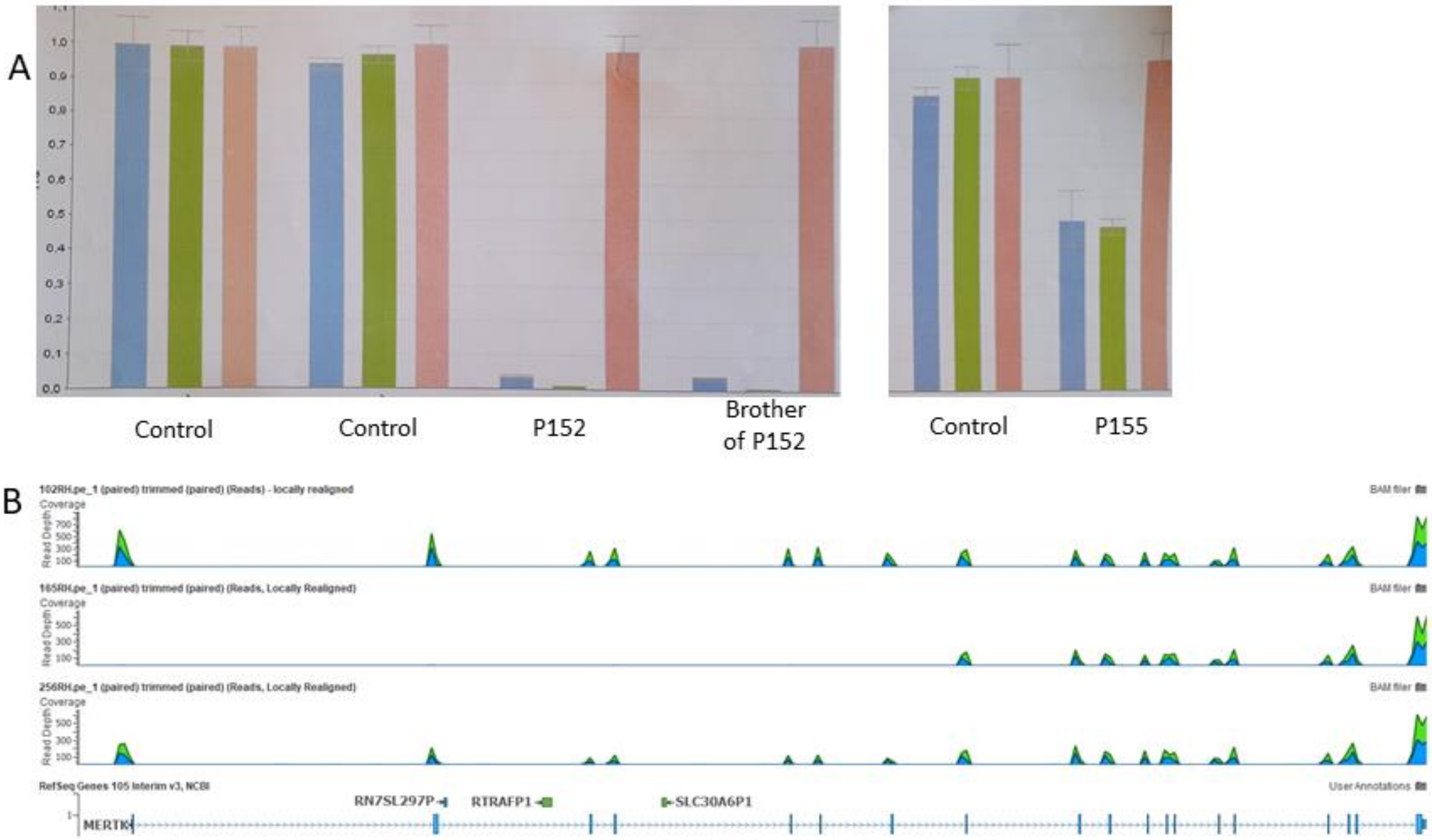

Figure S1

A) Results from qPCR analysis showing homozygogous deletion in P152 and his brother and heterozygous deletion in P155. Blue: exon 2, green: exon 4 and pink: exon 15.

B) Aligment of BAM files in Genome Browse showing top: control, middle: P155 (homozygous deletion) and bottom: P155 (heterozygous deletion).

Table S1

Gene	OMIM gene	Inheritance	Phenotype	Number of mutations in HGMD (2018.2)	Function	NM-number	Gene ID
ABCA4	601691	AR	Stargardt disease; retinitis pigmentosa 19	1030	Visual cycle	NM_000350.2	NG_009073.1
ADAM9	602713	AR	Cone-rod dystrophy	8	Cell adhesion/structure	NM_003816.2	NG_016335.1
ADGRA3	*612303*	AR	Retinal dystrophy	4	Signal transduction (G-coupled 7 TM receptor)	NM_145290.3	NG_032963.1
ADGRV1	602851	AR	Usher syndrome 2C	184	Signal transduction (G-coupled 7 TM receptor)	NM_032119.3	NG_007083.2
AIPL1	604392	AR	Leber congenital amaurosis 4	70	Nuclear transport, protein trafficking, chaperone activity	NM_014336.4	NG_008474.1
ALMS1	606844	AR	Alstrom syndrome	291	Cilia function	NM_015120.4	NG_011690.1
ARL2BP	*615407*	AR	Retinitis pigmentosa +/- situs inversus	4	Transcription (cofactor)	NM_012106.3	NG_033905.1
ARL6	608845	AR	Bardet-Biedl syndrome 3	21	Cilia function	NM_177976.2	NG_008119.2
BBIP1	*613605*	AR	Bardet-Biedl syndrome 18	1	Cilia function	NM_001195306.1	NG_041778.1
BBS1	209901	AR	Bardet-Biedl syndrome 1	94	Cilia function	NM_024649.4	NG_009093.1
BBS10	610148	AR	Bardet-Biedl syndrome 10	92	Cilia function	NM_024685.3	NG_016357.1
BBS12	610683	AR	Bardet-Biedl syndrome 12	50	Cilia function	NM_152618.2	NG_021203.1
BBS2	606151	AR	Bardet-Biedl syndrome 2	81	Cilia function	NM_031885.3	NG_009312.1
BBS4	600374	AR	Bardet-Biedl syndrome 4	47	Cilia function	NM_033028.4	NG_009416.2
BBS5	603650	AR	Bardet-Biedl syndrome 5	25	Cilia function	NM_152384.2	NG_011567.1

BBS7	607590	AR	Bardet-Biedl syndrome 7	38	Cilia function	NM_176824.2	NG_009111.1
BBS9	607968	AR	Bardet-Biedl syndrome 9	41	Cilia function	NM_198428.2	NG_009306.2
BEST1 (AD)	607854	AD	Macular dystrophy, vitelliform 2	292	Ion channel (Ca2+)	NM_004183.3	NG_009033.1
BEST1 (AR)	607854	AR	Bestrophinopathy		Ion channel (Ca2+)	NM_004183.3	NG_009033.1
C1QTNF5	608752	AD	Retinal degeneration late onset	6	Cell adhesion/structure	NM_015645.4	NG_012235.1
C21ORF2	*603191*	AR	Early onset retinal dystrophy	17	Cilia function	NM_004928.2	NG_032952.1
PCARE	613425	AR	Retinitis pigmentosa 54	40	Cilia function	NM_001029883.2	NG_021427.1
C8ORF37	*614477*	AR	Bardet-Biedl syndrome 21; Cone-rod dystrophy 16; Retintis pigmentosa 64	12	Cilia function	NM_177965.3	NG_032804.1
CA4	114760	AD	Retinitis pigmentosa 17	8	Acid overload removal	NM_000717.3	NG_012050.2
CABP4	608965	AR	Cone-rod synaptic disorder, congenital non progressive	11	Synaptic function. Ca2+ influx regulator	NM_145200.3	NG_021211.1
CACNA1F	300110	XL	Åland eye disease; Night blindness congenital stationary	170	Ion channel (Ca2+)	NM_005183.3	NG_009095.2
CACNA2D4	608171	AR	Retinal cone dystrophy	4	Ion channel (Ca2+)	NM_172364.4	NG_012663.1
CDH23	605516	AR	Usher type 1D; deafness	288	Cell adhesion/structure	NM_022124.5	NG_008835.1
CDH3	114021	AR	Hypotrichosis congenital with juvenile macular dystrophy; Ectodermal	29	Cell adhesion/structure	NM_001793.4	NG_009096.1
CDHR1	609502	AR	Retintis pigmentosa 65; Cone-rod dystrophy 15	34	Cell adhesion/structure	NM_033100.3	NG_028034.1
CEP290	610142	AR	Leber congenital amaurosis 10; Meckel syndrome 4; Senior Loken syndrome 6;	262	Cilia function	NM_025114.3	NG_008417.1

CERKL	608381	AR	Retinitis pigmentosa 26	34	No information	NM_001030311.2	NG_021178.1
CHM	300390	XL	Choroideremia	273	Signal transduction	NM_000390.2	NG_009874.2
CLRN1	606397	AR	Usher syndrome type 3A; Retinitis pigmentosa 61	37	No information	NM_174878.2	NG_009168.1
CNGA1	123825	AR	Retinitis pigmentosa 49	27	Phototransduction	NM_000087.3	NG_009193.1
CNGB1	600724	AR	Retinitis pigmentosa 45	34	Phototransduction	NM_001297.4	NG_016351.1
CNGB3	605080	AR	Achromatopsia 3	114	Phototransduction	NM_019098.4	NG_016980.1
CNNM4	*607805*	AR	Jalili syndrome	23	No information	NM_020184.3	NG_016608.1
CRB1	604210	AR	Leber congenital amaurosis 8; Retinitis pigmentosa 12	299	Cell adhesion/structure	NM_201253.2	NG_008483.2
CRX	602225	AD	Cone-rod dystrophia; Leber congenital amaurosis 7	92	Transcription factor	NM_000554.4	NG_008605.1
DHDDS	608172	AR	Retintis pigmentosa 59	8	No information	NM_024887.3	NG_029786.1
ELOVL4	605512	AD	Stargardt disease 3	11	Cell adhesion/structure	NM_022726.3	NG_009108.1
EYS	612424	AR	Retintis pigmentosa 25	252	Cell adhesion/structure	NM_001142800.1	NG_023443.2
FAM161A	613596	AR	Retintis pigmentosa 28	17	Cilia function	NM_001201543.1	NG_028125.1
FLVCR1	609144	AR	Ataxia, posterior column, with retintis pigmentosa	14	Transport (heme)	NM_014053.3	NG_028131.1
FSCN2	607643	AD	Reported retinitis pigmentosa 30	1	No information	NM_001077182.2	NG_015964.1
GNAT1	139330	AD	Night blindness congenital stationary 3	7	Phototransduction	NM_144499.2	NG_009831.1

GPR179	614615	AR	Night blindness congenital stationary	14	Signal transduction (G-coupled 7 TM receptor)	NM_001004334.3	NG_032655.2
GRK1	180381	AR	Oguchi disease 2	16	Phototransduction	NM_002929.2	
GRM6	604096	AR	Night blindness congenital stationary 1B	35	Signal transduction (glutamate receptor)	NM_000843.3	NG_008105.1
GUCA1A	600364	AD	Cone dystrophy 3; Cone-rod dystrophy 14	20	Phototransduction	NM_000409.3	NG_009938.1
GUCA1B	602275	AD	Retinitis pigmentosa 48	4	Phototransduction	NM_002098.5	NG_016216.1
GUCY2D (AD)	600179	AD	Cone-rod dystrophy 6 (AD)	219	Phototransduction	NM_000180.3	NG_009092.1
GUCY2D (AR)	600179	AR	Leber congenital amaurosis 1 (AR);		Phototransduction	NM_000180.3	NG_009092.1
IDH3B	604526	AR	Retinitis pigmentosa 46	0	Citric acid cycle	NM_006899.3	NG_012149.1
IMPDH1	146690	AD	Retinitis pigmentosa 10; Leber congenital amaurosis 11	15	Cell growth	NM_000883.3	NG_009194.1
IMPG2	607056	AR	Retinitis pigmentosa 56; Macular dystrophy	34	Cell adhesion/structure	NM_016247.3	NG_028284.1
INPP5E	*613037*	AR	Joubert syndrome 1	46	Signal transduction	NM_019892.4	NG_016126.1
IQCB1	609237	AR	Senior Loken syndrome	39	Cilia function	NM_001023570.2	NG_015887.1
KCNV2	607604	AR	Retinal cone dystrophy	87	Ion channel (K+)	NM_133497.3	NG_012181.1
KLHL7	611119	AD	Retinitis pigmentosa 42	11	No information	NM_001031710.2	NG_016983.1
LCA5	*611408*	AR	Leber congenital amaurosis 5	47	Cilia function	NM_181714.3	NG_016011.1
LRAT	604863	AR	Leber congenital amaurosis 14	16	Visual cycle	NM_004744.4	NG_009110.1

LRIT3	*615004*	AR	Night blindness congenital stationary	5	Cell adhesion/structure	NM_198506.4	NG_033249.1
LZTFL1	*606568*	AR	Bardet-Biedl syndrome 17	3	Cilia function	NM_020347.3	NG_033917.1
MAK	*154235*	AR	Retinitis pigmentosa 62	16	Cilia function	NM_001242957.1	NG_030040.1
MERTK	604705	AR	Retinitis pigmentosa 38	58	Phagocytosis	NM_006343.2	NG_011607.1
MKKS	604896	AR	Bardet-Biedl syndrome 6; McKusick Kaufmann syndrome	55	Cilia function	NM_018848.3	NG_009109.1
MKS1	*609883*	AR	Bardet-Biedl syndrome13; Joubert syndrome; Meckel syndrome	46	Cilia function	NM_017777.3	NG_013032.1
MVK	*251170*	AR	Retinitis pigmentosa	169	Signal transduction	NM_000431.3	NG_007702.1
MYO7A	276903	AR	Usher syndrome 1B; isolated deafness	468	Cilia function	NM_000260.3	NG_009086.1
NR2E3 (AD)	604485	AD	Retinitis pigmentosa 37 (AD)	69	Transcription factor	NM_014249.3	NG_009113.2
NR2E3 (AR)	604485	AR	Enhanced S-cone syndrome (AR)		Transcription factor	NM_014249.3	NG_009113.2
NRL	162080	AD	Retinitis pigmentosa 27	23	Transcription factor	NM_006177.3	NG_011697.1
NYX	300278	XL	Night blindness congenital stationary	87	No information	NM_022567.2	NG_009112.1
OFD1	*300170*	XL	Joubert syndrome 10	152	Cilia function	NM_003611.2	NG_008872.1
PCDH15	605514	AR	Usher syndrome 1F; isolated deafness	101	Cell adhesion/structure	NM_033056.3	NG_009191.2
PDE6A	180071	AR	Retinitis pigmentosa 43	38	Phototransduction	NM_000440.2	NG_009102.1
PDE6B (AD)	180072	AD	NBSC (AD)	103	Phototransduction	NM_000283.3	NG_009839.1

PDE6B (AR)	180072	AR	Retinitis pigmentosa 40 (AR)		Phototransduction	NM_000283.3	NG_009839.1
PDE6C	600827	AR	Cone dystrophy 4	36	Phototransduction	NM_006204.3	NG_016752.1
PDE6G	180073	AR	Retinitis pigmentosa 57	2	Phototransduction	NM_002602.3	NG_009834.1
PDZD7	612971	?	Usher syndrome 2C	18	Cilia function	NM_001195263.1	NG_028030.1
PITPNM3	608921	AD	Cone-rod dystrophy 5	3	Signal transduction (phosphaditylinositol transfer	NM_031220.3	NG_016020.1
PRCD	610598	AR	Retinitis pigmentosa 36	6	No information	NM_001077620.2	NG_016702.1
PROM1	604365	AR	Cone-rod dystrophy 12; Macular dystrophy 2; Retinitis pigmentosa 41 (1	58	Cellular structure	NM_006017.2	NG_011696.1
PRPF3	607301	AD	Retinitis pigmentosa 18	6	Splicing factor	NM_004698.2	NG_008245.1
PRPF31	606419	AD	Retinitis pigmentosa 11	142	Splicing factor	NM_015629.3	NG_009759.1
PRPF6	*613979*	AD	Retinitis pigmentosa 60	4	Splicing factor	NM_012469.3	NG_029719.1
PRPF8	607300	AD	Retinitis pigmentosa 13	37	Splicing factor	NM_006445.3	NG_009118.1
PRPH2 (RDS)	179605	AD + DIGEN (+ROM1)	Retinitis pigmentosa 7; Leber congenital amaurosis 18; Macualr	161	Cell adhesion/structure	NM_000322.4	NG_009176.1
RAB28	*612994*	AR	Cone-rod dystrophy 18	4	Intracellular trafficing	NM_004249.3	NG_033891.1
RAX2	610362	AD	Cone-rod dystrophy 11	4	Transcription	NM_032753.3	NG_011565.1
RBP3	180290	AR	?Retinitis pigmentosa 66	8	Visual cycle	NM_002900.2	NG_029718.1
RBP4	180250	AR	Retinal dystrophy plus colobom; microphthalmia plus colobom	7	Visual cycle	NM_006744.3	NG_009104.1

RDH12	608830	AR	Leber congenital amaurosis 13	92	Visual cycle	NM_152443.2	NG_008321.1
RDH5	601617	AR	Fundus albipunctatus	48	Visual cycle	NM_002905.3	NG_008606.1
RGR	600342	AR	Retinitis pigmentosa 44	8	Signal transduction (G-coupled 7 TM receptor)	NM_001012720.1	NG_009106.1
RGS9	604067	AR	Bradyopsia	2	Phototransduction	NM_003835.3	NG_013021.1
RGS9BP	607814	AR	Bradyopsia	6	Phototransduction	NM_207391.2	NG_016751.1
RHO	180380	AD	Retinitis pigmentosa 4	198	Phototransduction	NM_000539.3	NG_009115.1
RIMS1	606629	AD	Cone-rod dystrophy 7	4	Exocytosis	NM_014989.5	NG_016209.1
RLBP1	180090	AR	Rod-cone dystrophy; retinitis puctata albescens	31	Visual cycle	NM_000326.4	NG_008116.1
ROM1	180721	DIGEN (+PRPH	Retinitis pigmentosa 7	11	Disc morphogenesis	NM_000327.3	NG_009845.1
RP1 (AD)	603937	AD	Retinitis pigmentosa 1	153	Cilia function	NM_006269.1	NG_009840.1
RP1 (AR)	603937	AR	Retinitis pigmentosa 1		Cilia function	NM_006269.1	NG_009840.1
RP1L1	608581	AR	Occult macular dystrophy	27	Cilia function	NM_178857.5	NG_028035.1
RP2	300757	XL	Retinitis pigmentosa 2	106	Signal transduction	NM_006915.2	NG_009107.1
RP9	607331	AD	?Retinitis pigmentosa 9	0	Splicing factor	NM_203288.1	NG_012968.1
RPE65	180069	AR	Leber congenital amaurosis 2; Retinitis pigmentosa 20	178	Visual cycle	NM_000329.2	NG_008472.1
RPGR	312610	XL	Cone-rod dystrophy 3; Retinitis pigmentosa 3	192	Cilia function	NM_000328.2	NG_009553.1

RPGRIP1	605446	AR	Leber congenital amaurosis 6; Cone-rod dystrophy 13	122	Cilia function	NM_020366.3	NG_008933.1
SAG	181031	AR	Oguchi disease; Retinitis pigmentosa 47	9	Phototransduction	NM_000541.4	NG_009116.1
SDCCAG8	*613524*	AR	Bardet-Biedl syndrome 16; Senior Loken syndrome	17	Cilia function	NM_006642.3	NG_027811.1
SEMA4A	607292	AR	Cone-rod dystrophy 10; Retinitis pigmentosa 35	7	Cell-cell signalling	NM_022367.3	NG_027683.1
SLC24A1	603617	AR	Night blindness congenital stationary	6	Phototransduction	NM_004727.2	NG_031968.2
SNRNP200	611664	AD	Retinitis pigmentosa 33	21	Splicing factor	NM_014014.4	NG_018973.1
SPATA7	609868	AR	Leber congenital amaurosis 3	36	Cilia function	NM_018418.4	NG_021183.1
TEAD1	*189967*	AD	Sveinsson chorioretinal atrophy	2	Transcription factor	NM_021961.5	NG_0021302.1
TIMP3	188826	AD	Sorsby fundus dystrophy	17	Matrix metalloproteinase	NM_000362.4	NG_009117.1
TOPORS	609507	AD	Retinitis pigmentosa 31	13	Cilia function	NM_005802.4	NG_017050.1
TRIM32	602290	AR	?Bardet-Biedl syndrome 11	15	E3 ubiquitin ligase	NM_012210.3	NG_011619.1
TRPM1	603576	AR	Night blindness congenital stationary	65	Ion channel (Ca2+)	NM_002420.5	NG_016453.2
TTC8	608132	AR	Bardet-Biedl syndrome 8	13	Cilia function	NM_198309.3	NG_008126.1
TULP1	302280	AR	Leber congenital amaurosis 15; Retinitis pigmentosa 14	62	Cilia function	NM_003322.4	NG_009077.1
USH1C	605242	AR	Usher syndrome 1C; isolated deafness	38	Scaffolding	NM_005709.3	NG_011883.1
USH1G	607696	AR	Usher asynrome 1G	28	Scaffolding	NM_173477.4	NG_007882.2

USH2A	608400	AR	Usher syndrome 2A; Retinitis pigmentosa 39	974	Cilia function	NM_206933.2	NG_009497.1
WHRN	607928	AR	Usher type 1D; Deafness 31	21	Cilia function	NM_015404.3	NG_016700.1
ZNF513	613598	AR	?Retinitis pigmentosa 58	1	Transcription factor (possible)	NM_144631.5	NG_028219.1

Table S2

Primers used for qPCR

Exon/intron	Primer 1	Primer 2	Amplicon size (bp)
Exon 2	CAAACTGACCACACACCGCT	CTGGTACCCACTGGCGTGAG	51
Exon 4	CCTGAGAGCATGAATGTCACCA	CTGACAGGTGAGGTTGAAGGC	51
Intron 15	GCTCTGTCCCACTCTGAAGGA	AGGTATGAGGTCAAACGTGATCC	53

Primers used for breakpoint mapping

MERTK-IVS7-295-RH gaggggcaaacaacagatggcACATGGCCAGCTAGAGATCA

MERTK-del-FH3 AcccactgcttactggcttatcGGCAGATCACCAGAGATTAGGA
